# Transseptal access: A review of contemporary tools

**DOI:** 10.1111/jce.15428

**Published:** 2022-03-08

**Authors:** Rachel M. Kaplan, Jeremiah Wasserlauf, Bradley P. Knight

**Affiliations:** ^1^ Section of Electrophysiology, Division of Cardiology, Feinberg School of Medicine Northwestern University Chicago Illinois USA; ^2^ Section of Electrophysiology, Division of Cardiology Rush University Chicago Illinois USA

**Keywords:** intracardiac echocardiography, radiofrequency needle, radiofrequency wire, transseptal

## Abstract

Transseptal left atrial catheterization is routinely used for many common catheter‐based interventions. Tools for transseptal catheterization have advanced over the recent years. Such tools include imaging advances with intracardiac echocardiology as well as an array of needles, wires, and dilators to achieve transseptal access with greater ease and safety. This study will discuss the contemporary tools for transseptal catheterization and guidance for difficult cases.

## 1 INTRODUCTION

Transseptal left atrial catheterization was first described by Ross and Braunwald over 60 years ago.^1^ Since then, left atrial catheter‐based interventions have become widespread with pulmonary vein isolation and left atrial appendage occlusion procedures now common in the management of atrial fibrillation. Over the years, new tools have been developed to facilitate transseptal catheterization and improve the safety of the procedure. This study will describe the contemporary tools available for transseptal catheterization and guidance for difficult cases.

### 1.1 Imaging

**Figure 1:**
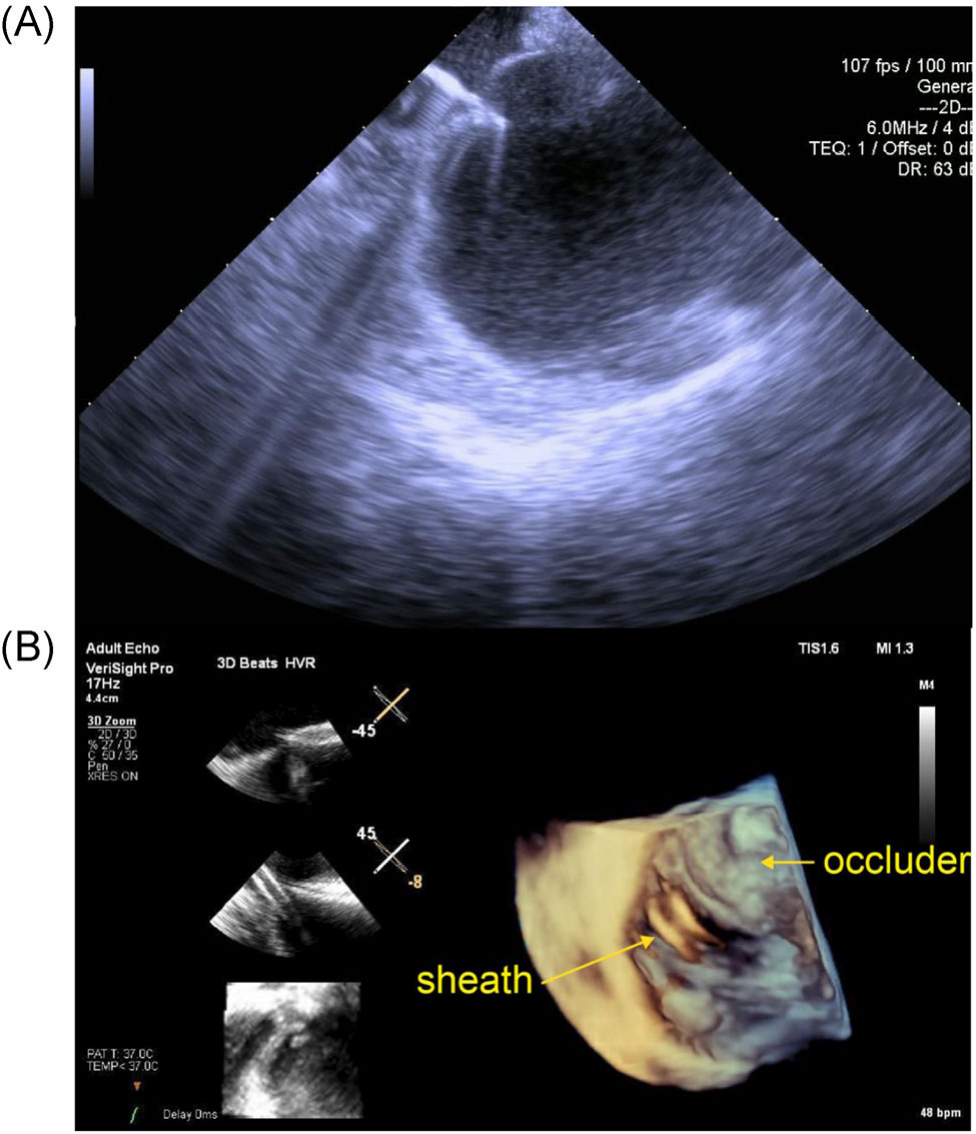
Images of the fossa ovalis with transseptal needle tenting the septum (A) and of 3D image of transseptal access below an atrial septal defect closure device (B)

Perhaps the greatest advance in transseptal catheterization is intracardiac echocardiography (ICE). ICE enables the operator to view the exact position of the transseptal equipment within the heart rather than relying on general positioning by fluoroscopy.^2^ With the ICE catheter in the right atrium, the interatrial septum can be directly viewed and nearby structures including the aorta can be seen with simple rotations of the catheter to confirm that the selected position for transseptal catheterization is a safe one. Standard two‐dimensional ICE images can provide clear visualization of the transseptal needle and sheath as shown in Figure 1A. The needle is shown to tent the septum (Figure 1A) before puncturing through to the left atrium. Newer ICE technologies can incorporate three‐dimensional imaging and facilitate more difficult transseptal catheterization procedures.^3^ An example of such imaging used for transseptal catheterization below a pre‐existing atrial septal defect closure device is shown in Figure 1B. ICE has also enabled advances in fluoroscopy reduction and some operators perform transseptal catheterizations as well as the remainder of left atrial ablations without the use of any fluoroscopy.

### 1.2 Contemporary tools for transseptal catheterization

Tools for transseptal catheterization require a needle or needle‐based technology for crossing the interatrial septal tissue and a support device (e.g., sheaths) to enable the needle to reach the appropriate location and provide support for the crossing. Some contemporary tools employ radiofrequency to assist with crossing the septum. Other operators have electrified standard needles or wires using common electrocautery equipment. These approaches will be described in this study and illustrated from in‐vitro demonstrations using porcine atrial tissue, corkboard, and a saline bath.

### 1.3 Transseptal needles—nonradiofrequency

**Figure 2:**
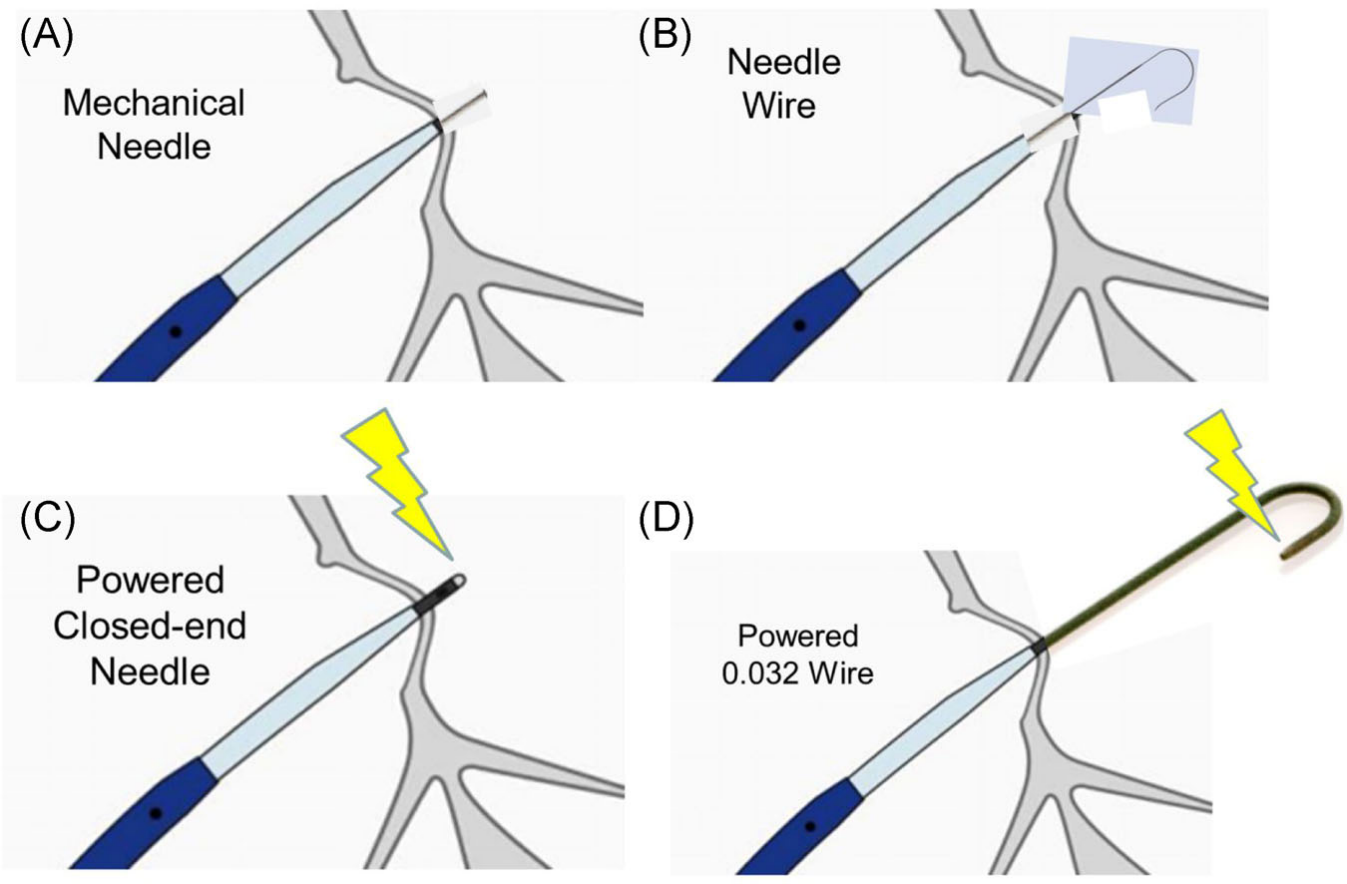
Mechanical transseptal tools (needle—A, needle on wire—B) and radiofrequency assisted transseptal tools (needle—C, needle on wire—D)

Early needles for transseptal catheterization were developed by Brockenbrough in 1962.^4^ These evolved to the present time with the BRK needle based on a similar platform. The BRK™ needle (Abbott) consists of a stiff needle with a stylet (Figure 2A). The stylet prevents the needle from the scraping the inner lumen of the transseptal sheath during needle advancement. The stylet is then removed, and the inner lumen port attached to a pressure line. The BRK needle comes in multiple pre‐shaped curves and several lengths. The BRK needle is sharp and crosses the septum by mechanical force alone.

**Figure 3:**
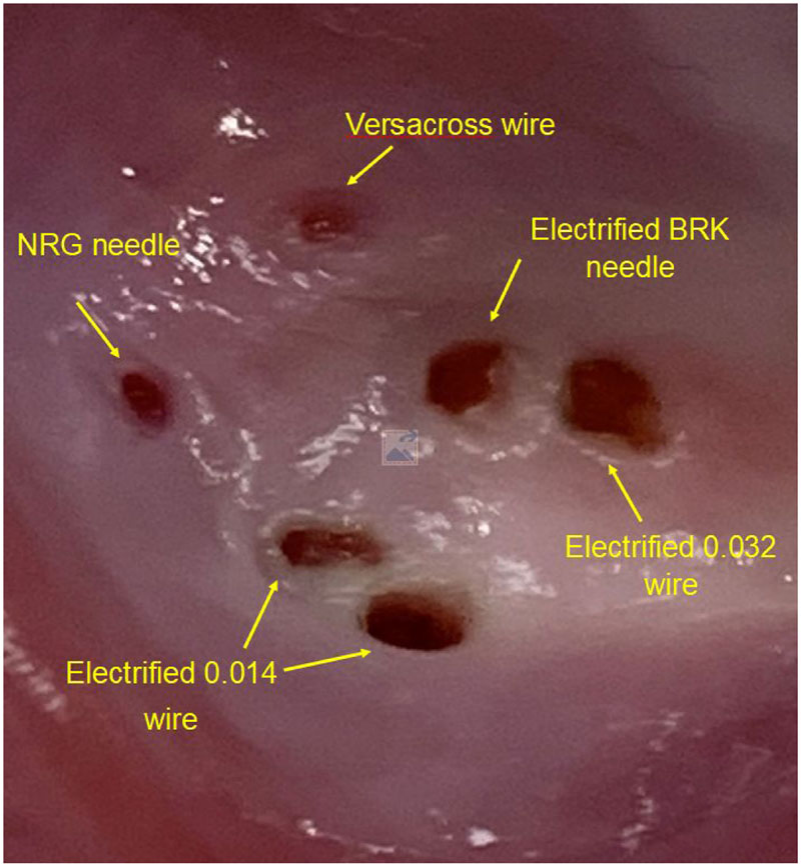
Transseptal puncture sites from various technologies in an in vitro porcine atrial model

Some operators have combined the idea of radiofrequency assisted transseptal catheterization with the traditional Brockenbrough‐type needle by applying a standard electrocautery pen to the end of the metal needle.^5^ Crossing atrial tissue on the in‐vitro model by applying 50 Watts from a standard electrocautery pen to the end of a BRK needle is shown in Video S1. The resulting puncture site can be seen in Figure 3. While this technique may be feasible and appear cost‐effective, septal coring and potential embolism can occur due to differences in how the tissue is heated.^6^ Standard electrocautery equipment can deliver high power over a longer time period which raises the local tissue temperature gradually and destroys tissue by coagulative necrosis. Forceful advancement of tools through coagulated tissue may liberate particles and particularly with hollow tools, core out pieces of the septum.^7^, ^8^ These particles can embolize and cause major complications. In contrast, the generator used specifically for radiofrequency transseptal equipment utilizes a high‐energy electric field that instantaneously heats the tissue to 100°C. Consequently, there is perforation at the catheter tip but minimal thermal damage to surrounding tissue.^7^ Additionally, the system automatically titrates the radiofrequency energy needed based on the tissue variance and so the minimum power needed to perforate the tissue is delivered. If even rare transient ischemic attacks or cardiac tamponade can be avoided with specialized radiofrequency transseptal equipment, it is conceivable to offset the modest added costs for these tools.^9^

Unlike the large open‐end of Brockenbrough‐type needles, the NRG has small side holes for irrigation but no large central hole, which avoids coring the septal tissue. Because of this risk, it is our approach to avoid applying electrocautery to transseptal needles that are not designed for radiofrequency support. An example of an electrified BRK needle both melting the tip of the dilator and coring tissue is shown in Video S2. When the needle is flushed, the cored tissue is released and can be clearly seen in close‐up imaging (Video S3).

### 1.4 Transseptal needles—radiofrequency

A radiofrequency transseptal needle (NRG®) was developed by Baylis Medical and its use was first described in 2010.^10^, ^11^ The NRG also comes in multiple pre‐shaped curves and several lengths and is sufficiently malleable to adjust the curve to the operator's preference. The NRG has an inner lumen port and an electrical connector (Figure 2B). The electrical connector is connected to the Baylis generator via a cable. The tip of the NRG is more blunt than the BRK needle tip. When positioned at the optimal location for transseptal access, radiofrequency energy is applied (standard setting is 10 Watts for 1–2 s), facilitating passage of the needle across the septum. Minimal mechanical force is required due to the assistance of radiofrequency energy. An example of this radiofrequency needle crossing tissue in the in vitro model is shown in Video S4, with the resulting puncture site demonstrated in Figure 3. The NRG has been demonstrated to increase first attempt success in transseptal catheterization as well as shorter instrumentation time and even a lower risk of pericardial tamponade as compared to the BRK needle.^12^ However, in comparison to the BRK needle, the NRG is more expensive. The NRG tip can also be marked on some electrophysiology mapping systems by changing a switch on the generator right before crossing the septum. Marking the site on the mapping system can be a helpful way to reduce fluoroscopy usage in the event of needing to repeat a transseptal catheterization due to loss of access or as a way to confirm position with other catheters during the procedure, relative the transseptal location.

### 1.5 Transseptal needle based technologies—wires

After a needle is used to cross the interatrial septum, the sheath and dilator are then advanced over the needle. The needle is then exchanged for a j‐tip wire which is positioned in the left superior pulmonary vein or a pigtail‐shaped spring coil wire positioned in the left atrium to support safe passage of the sheath into the left atrium. Alternatively, the sheath can be exchanged for a different sheath as required by the procedure. This transition requires careful management of the transseptal equipment to avoid loss of position and to ensure there is no entry of air into the left atrium. Additionally, this exchange requires multiple steps after crossing the interatrial septum before the operator is ready to proceed with the rest of the planned left atrial procedure. For these reasons, there was interest in developing an all‐in‐one wire‐based needle tool for transseptal catheterization that can act as both the needle and wire. There are two types of wire‐based needle tools available: one that involves radiofrequency, and one that does not.

The SafeSept® transseptal guidewire (Pressure Products) is a wire with a sharp needle tip at the end (Figure 2C). When straightened by placement with a sheath and dilator, the sharp needle is at the leading edge. Once advanced past the puncture site and no longer supported by the dilator, the tip of the wire assumes a J position so that the sharp needle is no longer the leading edge. The wire can then be advanced into the left superior pulmonary vein to support the sheath crossing. This wire was studied during cryoballoon pulmonary vein isolation procedures for atrial fibrillation and found to decrease procedure time as compared with the traditional needle approach.^13^

### 1.6 Transseptal needle based technologies—radiofrequency wires

The Versacross® (Baylis Medical) wire incorporates a radiofrequency needle tip on a wire (Figure 2D). Similar to the tip of the NRG needle, the tip of the Versacross® is relatively blunt. The opposite end of the wire is connected to the same Baylis generator as the NRG needle would be. The general principle is similar to the SafeSept® wire—when the tip is just outside the dilator, the wire remains straight, and the tip is used as the needle to puncture the septum. Once the tip passes through, it is advanced without the support of the dilator and assumes an atraumatic J position allowing further advancement into the left superior pulmonary vein to support the sheath. Recent studies of the Versacross® have demonstrated increased efficiency in the left atrial workflow.^14^ The Versacross® is also available in both the J tip and a pigtail tip (Figure 2D). The latter curves to a pigtail (similar in size to a 5 or 6 French pigtail catheter) after it is advanced beyond the support of the sheath. Perhaps the most significant barrier to widespread use of the Versacross® at present remains the increased cost with the newer system. A demonstration of the Versacross® in the in vitro model is shown in Video S5 with the resulting puncture site in Figure 3.

### 1.7 Transseptal technologies—electrifying nonneedle guidewires

Similar to the strategy of applying an electrocautery pen to a traditional Brockenbrough‐type needle, some operators have employed this technique on standard guidewires. However, in comparison to radiofrequency wires like the Versacross®, such a strategy was less likely to be effective at crossing the interatrial septum and was associated with greater risks of damage to the other equipment such as melting or deforming the dilator or causing more tissue damage than planned.^8^ Examples of electrifying a 0.032 guidewire and an 0.014 guidewire to cross in the in vitro model are shown in Videos S6 and S7, respectively. The puncture sites can be seen in Figure 3.

### 1.8 Transseptal technologies – integrated needle/dilator

**Figure 4:**
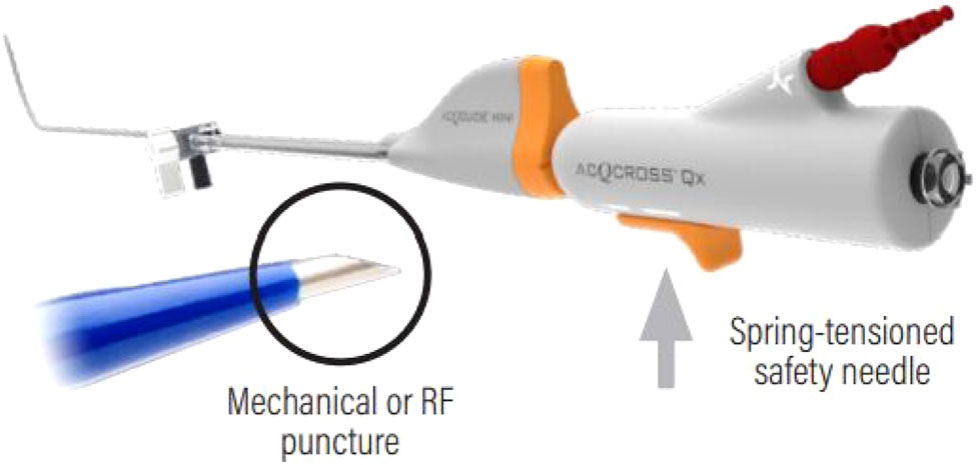
AcQcross™ integrated needle/dilator system (https://www.acutusmedical.com/media/Acutus-Medical-Transseptal-Product-Portfolio-MM-67-Rev-G.pdf)

In contrast to the wire‐based needle systems, Acutus Medical has developed a needle that is integrated with its dilator (AcQcross™). The dilator is advanced over a standard guidewire and can be placed through either an Acutus Medical sheath or another sheath of the operator's choosing. When positioned at the septum, the needle is deployed via a spring‐loaded system (Figure 4). The needle can be used with or without radiofrequency assistance. After crossing the septum, the guidewire is advanced, and the needle is retracted without requiring any exchange of the system.

### 1.9 Transseptal sheaths

There are multiple types of standard fixed‐curve sheaths that are commonly used for transseptal catheterization. These include the classic Mullins sheath (Medtronic) that was used during early experience with transseptal access as well as the Schwartz (SL) series (Abbott) and Preface® sheaths (Biosense Webster). Additionally, several companies that make transseptal needle technologies have their own line of fixed‐curve sheaths that could be used as well. Steerable sheaths may also be used, though typically this selection is based more on the need for steerable design for the anticipated left atrial procedure rather than needing steerability for the transseptal catheterization itself.

Baylis Medical has also developed a line of large bore transseptal access dilators that do not require a sheath for support. These dilators are made of a malleable material that enables the operator to mold them to the desired curve. The dilator reaches a maximal dimension of 12 French, and so can be used to dilate the transseptal site before exchanging for another large bore access sheath (such as the 14 French cryoballoon sheath or Watchman delivery sheath).

### 1.10 Difficult cases—crossing the septum

In cases where the interatrial septum is hypertrophied or scarred from prior transseptal procedures, crossing with a nonradiofrequency needle can prove difficult. Even with a radiofrequency needle, the septum can be sufficiently stiff that it is difficult for the tip of the dilator to cross far enough to then exchange the needle for the guidewire. In such cases, the needle‐in‐wire tools can be more helpful as once across the septum, the wire can continue to be advanced into the left superior pulmonary vein to support the sheath crossing.

### 1.11 Difficult cases—dilating the septum

**Figure 5:**
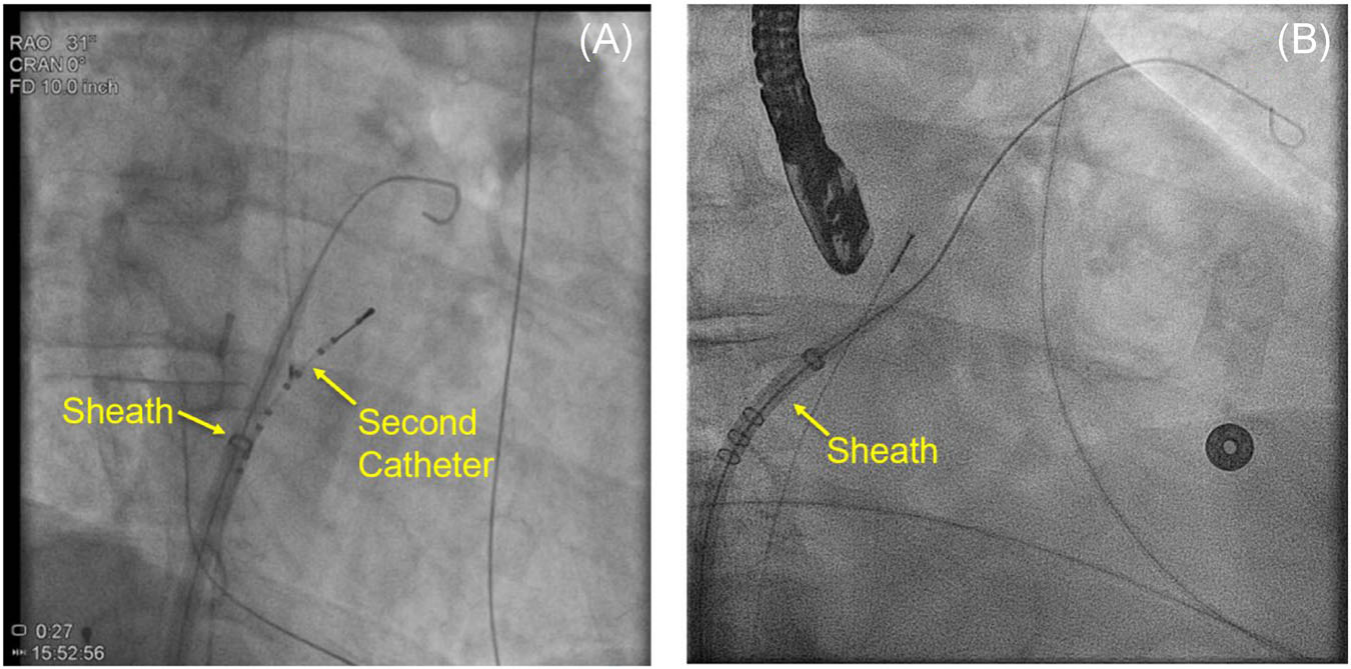
Shoehorn technique for crossing difficult septum. Second catheter advanced into left atrium for dilation (A); the second catheter has been withdrawn and the sheath advanced into the left atrium (B)

Dilating the transseptal puncture site sufficiently to allow the sheath to cross can be challenging, particularly in cases of hypertrophied septa or in individuals who have scar tissue from prior transseptal procedures. In these cases, using a second catheter to dilate the site can assist with the sheath crossing. To perform this “shoehorn” technique, the transseptal sheath is backed away from the site initially. A second catheter, often an electrophysiology catheter that may already be in the body or the ICE catheter, is advanced through the transseptal site by following the wire fluoroscopically. The transseptal sheath is then brought up to the transseptal opening. As forward pressure is applied to the transseptal sheath, the secondary catheter is withdrawn into the right atrium. This movement enables the transseptal sheath to pass into the left atrium (Figure 5). Alternatively, a separate large bore tapered dilator can be used to dilate the transseptal site, though this requires an additional exchange to then advance the planned left atrial access sheath.

## 2 CONCLUSION

Contemporary tools for transseptal catheterization have advanced considerably over the past few decades. Radiofrequency needles and wire‐based technologies have improved the safety and efficiency of transseptal catheterization. Widespread use of such tools may be limited by their increased cost.

## Supporting information

Video S1. Electrified BRK needle crossing atrial tissue.

Video S2. After electrification of BRK needle, the tip of the dilator is melted and cored tissue is released.

Video S3. Cored tissue flushed from the BRK needle after electrified crossing.

Video S4. Radiofrequency needle (NRG®) crossing atrial tissue.

Video S5. Radiofrequency wire (Versacross®) crossing atrial tissue.

Video S6. Electrification of an 0.032” guidewire to cross atrial tissue.

Video S7. Electrification of an 0.014” guidewire to cross atrial tissue.

## Data Availability

Data sharing is not applicable to this article as no new data were created or analyzed in this study.
